# Reliability of the TekScan MatScan^® ^system for the measurement of plantar forces and pressures during barefoot level walking in healthy adults

**DOI:** 10.1186/1757-1146-3-11

**Published:** 2010-06-18

**Authors:** Gerard V Zammit, Hylton B Menz, Shannon E Munteanu

**Affiliations:** 1 Musculoskeletal Research Centre, Faculty of Health Sciences, La Trobe University, Victoria 3086, Australia; 2 Department of Podiatry, Faculty of Health Sciences, La Trobe University, Victoria 3086, Australia

## Abstract

**Background:**

Plantar pressure systems are increasingly being used to evaluate foot function in both research settings and in clinical practice. The purpose of this study was to investigate the reliability of the TekScan MatScan^® ^system in assessing plantar forces and pressures during barefoot level walking.

**Methods:**

Thirty participants were assessed for the reliability of measurements taken one week apart for the variables maximum force, peak pressure and average pressure. The following seven regions of the foot were investigated; heel, midfoot, 3^rd^-5^th ^metatarsophalangeal joint, 2^nd ^metatarsophalangeal joint, 1^st ^metatarsophalangeal joint, hallux and the lesser toes.

**Results:**

Reliability was assessed using both the mean and the median values of three repeated trials. The system displayed moderate to good reliability of mean and median calculations for the three analysed variables across all seven regions, as indicated by intra-class correlation coefficients ranging from 0.44 to 0.95 for the mean and 0.54 to 0.97 for the median, and coefficients of variation ranging from 5 to 20% for the mean and 3 to 23% for the median. Selecting the median value of three repeated trials yielded slightly more reliable results than the mean.

**Conclusions:**

These findings indicate that the TekScan MatScan^® ^system demonstrates generally moderate to good reliability.

## Background

During functional activities such as walking, the human foot exerts a force upon the underlying surface, and in turn, a force of equal magnitude and opposite direction is exerted upon the foot. This force is commonly termed the ground reaction force [1,2]. Technological advances in pressure-sensing technology, enabling the quantification of the vertical component of this force and the contact area at different regions under the foot, have become commercially available for research and clinical applications. This has enabled further insight into the plantar loading characteristics of the foot during functional activities such as walking and running [3,4].

Elevated plantar pressures have been widely recognised as a causative factor in the development of several pedal pathologies, including the development of stress fractures [5], plantar calluses [6,7] and neuropathic ulceration [8]. Factors shown to be associated with elevated plantar pressures include forefoot deformity [9], increased heel pad stiffness [10] and lesser toe deformity [11]. The analysis of plantar forces and pressures has also played an integral role in the management of lower limb disorders. Specifically, footwear modifications [12] and redistributive insoles [13] aimed at offloading areas of high pressure prone to ulceration have been assessed for effectiveness in patients with diabetic peripheral neuropathy.

Commercially available systems currently employed by clinicians and researchers to assess dynamic plantar pressures include in-shoe measurement systems (Novel Pedar^®^, TekScan F-Scan^®^, RS-Scan Insole^® ^and IVB Biofoot^®^) and platform systems (Novel Emed^®^and the RS-Scan Footscan^®^) [14]. The validity of these measurement systems has been documented throughout the literature, suggesting they are able to accurately quantify dynamic plantar loading patterns of the foot [15-19]. Validity of the TekScan MatScan^® ^system has been reported by the manufacturer, displaying a mean percentage difference of 1.9% when compared against an AMTI force platform (TekScan Incorporated, personal communication, 26/02/2010) and has also been shown to be highly accurate in an independent study which compared several commonly used plantar pressure measurement systems [20]. However, to the authors knowledge, no study to date has investigated the reliability of the TekScan MatScan.^® ^ As this system is widely utilised by researchers and clinicians it is essential that its reliability is adequately established.

Therefore, the primary aim of this study was to determine the reliability of the TekScan MatScan^® ^system in assessing plantar forces and pressures during level barefoot walking using a test-retest analysis of thirty healthy asymptomatic participants. The secondary objective of this study was to determine if the calculation of median or mean values of plantar pressure and forces yielded more reliable measurements between trials.

## Methods

### Participants

Thirty participants (n = 30) were recruited for assessment from a university population. Participants included in the study were healthy asymptomatic adults, aged between 18 and 40 years of age. The Human Studies Ethics Committee at La Trobe University, Victoria, Australia provided ethical approval for the study (FHEC07/08). Written informed consent was obtained from all participants prior to data collection.

Age (years), gender, height (cm), weight (kg), body mass index (BMI) (kg/m^2^), and foot posture using the 6 item Foot Posture Index (FPI-6) were determined for each of the study participants at baseline. The FPI-6 was applied by one of the raters (GVZ) to quantify participant foot posture as being either pronated, neutral, or supinated [21]. This clinical assessment tool has been previously shown to be a valid indicator of arch structure from foot radiographs [22]. Reference values for interpretation of results are as follows; -12 to -5 highly supinated, -4 to -1 supinated, 0 to 5 normal, 6 to 9 pronated and, 10 to 12 highly pronated [23].

### Measurement apparatus

Plantar forces and pressures were recorded during level barefoot walking using the TekScan MatScan^® ^system (Boston, MA, USA). The system consists of a 5 mm thick floor mat (432 × 368 mm), comprising of 2288 resistive sensors (1.4 sensors/cm^2^), and sampling data at a frequency of 40 Hertz (Hz).

### Procedure

The two-step gait initiation protocol was used to capture dynamic plantar pressures, as it displays similar re-test reliability to the commonly used midgait protocol, however requires fewer trials [24-26]. The two step method involves striking the platform on the second step once a constant velocity has been reached, and is suggested to reproduce plantar force and pressure data that is reflective of foot function during gait. Trials were excluded and repeated if the plantar pressure recording was not satisfactorily positioned, the participant paused on the mat whilst walking, or if the participant did not continue to walk past the mat for more than two steps. Three trials of the left foot were recorded for each participant, as this number of trials has previously been found to be sufficient in ensuring adequate reliability of force and pressure data [27,28]. Plantar force and pressure measurements were recorded at baseline, and repeated at follow up one week later. A one week duration between sessions was chosen to ensure participants' gait characteristics remained reasonably consistent.

Maximum force, peak pressure and average pressure were the parameters measured in this study at seven regions of the foot. These three variables were assessed as they are the standard outputs of the MatScan^®^ system, and peak plantar pressure in particular has been found to be of importance in the development of pathological foot problems such as ulceration [29] and osteoarthritis [30], and determining the efficacy of treatment modalities such as redistributive insoles [31] and therapeutic footwear [32]. We used a mask with seven regions (heel, midfoot, 1^st ^MPJ, 2^nd ^MPJ, 3^rd^-5^th ^MPJs, hallux and lesser toes) to provide detailed information regarding the independent function of different segments of the foot. We have previously used this mask to examine age-related changes in foot function [33], clinical predictors of plantar loading in older people [34], and differences in plantar loading in people with osteoarthritis of the 1^st ^MPJ [35] and midfoot [30].

### Data processing

**Figure 1:**
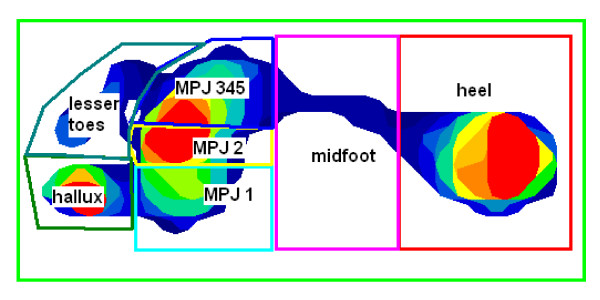
**An example of a typical walking trial produced by the TekScan MatScan^® ^system, displaying the seven masked regions used during analysis**.

Following data collection, Research Foot^® ^Version 5.24 was used to construct seven individual "masks" to determine maximum force (N), peak pressure (kPa) and average pressure (kPa) under the following regions of the foot: heel, midfoot, 3^rd^-5^th ^metatarsophalangeal joints (MPJ345), 2^nd ^metatarsophalangeal joint (MPJ2), 1^st ^metatarsophalangeal joint (MPJ1), hallux and the lesser toes (Figure 1). An overall 'total' was also calculated for the entire plantar surface area. To determine the reliability of reapplying the masks between sessions, the primary investigator (GVZ) constructed masks for 10 randomly selected participants, calculated maximum force values for all seven regions of interest, and repeated the process one week later without reference to the previous data.

### Statistical analysis

Statistical analysis was conducted using SPSS Version 14 for Windows (SPSS, Inc., Chicago, IL, USA). Prior to inferential analysis all data were explored for normality.

To maintain independence of data only the left foot of each participant was chosen to be assessed [36]. Reliability of mask application was assessed using intraclass correlation coefficients (ICCs). Intra-session reliability was assessed via the calculation of coefficients of variation (CVs) and ICCs across the three repeated trials within the same session. The analysis of absolute reliability provides information regarding within trial variability expressed as a percentage. Inter-session reliability was evaluated using both relative reliability statistics (ICCs) and absolute reliability statistics (mean differences, CVs, minimal detectable change [MDC] and 95% limits of agreement [95% LoAs]) for (i) using the mean of three trials, and (ii) using the median of three trials. Calculation of the mean occurred by summating the values, then dividing by the number of trials (3), whereas the median was defined as the middle value of the three captured trials. The median was calculated along with mean to deal with any data which may potentially be skewed.

First, to assess for systematic differences between sessions, paired *t-*tests were used to compare mean and median values of maximum force, peak pressure and average pressure for each individual region of the left foot. Second, to assess relative reliability between sessions ICCs (type 3, 1) were used. Interpretation of the ICCs was conducted in accordance with suggestions of Portney and Watkins [37], whereby values >0.75 indicate good reliability, values ranging from 0.50 to 0.75 imply moderate reliability and values <0.50 suggest poor reliability. Finally, to assess absolute reliability between sessions, CVs were calculated, providing information regarding between-trial variability expressed as a percentage and enabling direct comparisons between variables measured in different units. The MDC, also referred to as the smallest real difference, was calculated to provide an indication of the change in score necessary to assume a real change has occurred [38], and 95% LoAs were calculated to provide lower and upper limits within which the examiner can be 95% confident that the true score lies [39]. If differences between baseline and follow-up measurements were found, Cohen's *d *was calculated to determine the magnitude of these differences. Cohen's *d *is defined as the difference between 2 means divided by the pooled standard deviation for the baseline and follow-up values [40].

## Results

### Participant characteristics

Information describing participant characteristics is presented in Table 1. The overall mean age (SD) of participants was 28.2 (6.1) years (range 18 to 39 years) and the mean BMI was 23.7 (3.0) kg/m^2^. Males comprised 60% (n = 18) of the sample and participants exhibited a relatively normal foot posture, as evidenced by a mean FPI score of 4.3 (3.3) [23].

**Table 1:** Participant characteristics.

Variable	
Age (years) - mean (SD)	28.2 (6.1)
Gender (Male/Female)	18 M/12 F
Height (cm) - mean (SD)	174.0 (7.9)
Weight (kg) - mean (SD)	72.1 (12.0)
Body mass index (kg/m^2^) - mean (SD)	23.7 (3.0)
Foot Posture Index (FPI-6) - mean (SD)	4.3 (3.3)

### Reliability of mask application

Intraclass correlation coefficient values demonstrated good reliability with values ranging from 0.96 to 1.00 (Table 2).

**Table 2:** Intra-rater reliability of mask application, assessed with the variable maximum force.

Region	ICC (95% CI)
Total	1.00 (1.00 -1.00)
Heel	0.99 (0.98 - 0.99)
Midfoot	0.99 (0.98 - 0.99)
MPJ345	0.96 (0.85 - 0.99)
MPJ2	0.99 (0.96 - 0.99)
MPJ1	0.99 (0.96 - 0.99)
Hallux	0.99 (0.98 - 0.99)
Lesser toes	0.99 (0.97 - 0.99)

### Intra-session repeatability

Coefficients of variation and ICCs across the three repeated trials within the one session ranged from 3 to 22% and 0.83 to 0.98 respectively for maximum force, 3 to 32% and 0.65 to 0.92 for peak pressure, and 6 to 27% and 0.49 to 0.98 for average pressure (Table 3). The midfoot region demonstrated the largest variation between trials for all three parameters.

**Table 3:** Intra-session reliability (coefficients of variation [CoV], and intraclass correlation coefficients [ICC]) obtained from three repeated trials.

Region	Maximum force		Peak pressure		Average pressure	
	CoV (%)	ICC (95% CI)	CoV (%)	ICC (95% CI)	CoV (%)	ICC (95% CI)
Total	3.4	0.98 (0.96 - 0.99)	3.5	0.92 (0.85 - 0.96)	5.9	0.98 (0.96 - 0.99)
Heel	4.7	0.97 (0.94 - 0.98)	6.9	0.90 (0.83 - 0.95)	12.9	0.66 (0.37 - 0.83)
Midfoot	22.1	0.96 (0.93 - 0.98)	31.7	0.69 (0.45 - 0.84)	27.4	0.49 (0.06 - 0.74)
MPJ345	16.1	0.83 (0.70 - 0.91)	10.8	0.82 (0.63 - 0.91)	11.8	0.91 (0.84 - 0.95)
MPJ2	15.0	0.75 (0.54 - 0.87)	5.9	0.91 (0.84 - 0.95)	11.9	0.78 (0.60 - 0.89)
MPJ1	19.2	0.72 (0.49 - 0.86)	17.2	0.84 (0.71 - 0.92)	14.4	0.75 (0.53 - 0.87)
Hallux	15.2	0.86 (0.75 - 0.93)	13.7	0.85 (0.73 - 0.92)	15.0	0.88 (0.79 - 0.94)
Lesser toes	21.1	0.92 (0.83 - 0.95)	25.1	0.65 (0.35 - 0.82)	15.6	0.88 (0.78 - 0.94)

### Inter-session reliability - maximum force

The relative reliability between sessions when using the mean of three measurements was good for the total area, heel, midfoot, MPJ2, MPJ1 and lesser toes, as evidenced by ICCs ranging from 0.76 to 0.95, and moderate for MPJ345 and the hallux (ICCs of 0.72 and 0.71, respectively). The relative reliability between sessions when using the median of three measurements was good for all seven regions, as evidenced by ICCs ranging from 0.79 to 0.97 (Table 4).

**Table 4:** Inter-session reliability of maximum force (N).

	Mean of three trials					
**Region**	**Session 1** **mean (SD)**	**Session 2** **mean (SD)**	**ICC (95% CI)**	**CV (%)**	**MDC**	**95% LoA**

Total	665.38 (113.56)	654.50 (121.90)	0.92 (0.84 - 0.96)	5.1	91.01	-84.83 - 101.40
Heel	400.50 (70.21)	391.09 (88.16)	0.87 (0.75 - 0.94)	6.9	71.78	-66.69 - 85.51
Midfoot	111.31 (61.49)	108.56 (63.06)	0.95 (0.89 - 0.97)	13.2	39.72	-37.46 - 42.95
MPJ345	215.35 (56.88)	214.86 (52.07)	0.72 (0.49 - 0.85)	13.5	79.53	-80.02 - 81.10
MPJ2	149.94 (28.05)	147.98 (31.87)	0.76 (0.56 - 0.88)	9.9	40.01	-39.03 - 42.86
MPJ1	141.41 (41.58)	145.43 (36.19)	0.80 (0.61 - 0.90)	12.3	47.56	-52.86 - 44.72
Hallux	96.79 (26.38)	94.83 (25.40)	0.71 (0.47 - 0.85)	14.6	38.54	-36.97 - 40.80
Lesser toes*	52.56 (25.79)	58.55 (27.56)	0.92 (0.84 - 0.96)	5.1	24.52	-30.79 - 18.73

	**Median of three trials**					

Total	660.58 (119.64)	661.07 (115.03)	0.97 (0.94 - 0.99)	3.0	54.13	-54.92 - 55.80
Heel	393.54 (68.94)	397.86 (80.02)	0.91 (0.81 - 0.95)	5.8	59.23	-67.57 - 58.94
Midfoot	105.91 (62.17)	114.35 (65.51)	0.93 (0.85 - 0.96)	15.8	47.46	-56.88 - 39.81
MPJ345	218.09 (59.62)	215.45 (57.57)	0.81 (0.64 - 0.91)	11.8	70.12	-68.16 - 73.55
MPJ2	147.20 (29.81)	141.80 (31.68)	0.79 (0.62 - 0.89)	9.3	37.95	-42.76 - 33.93
MPJ1	141.02 (36.68)	141.12 (40.40)	0.79 (0.60 - 0.89)	12.5	48.25	-49.13 - 49.03
Hallux	98.48 (26.06)	93.65 (25.89)	0.78 (0.58 - 0.89)	12.8	33.93	-29.22 - 39.03
Lesser toes*	58.45 (30.90)	53.05 (27.07)	0.89 (0.78 - 0.95)	17.2	25.50	-21.08 - 32.07

* significant difference (p < 0.05) between session 1 and 2

The absolute reliability of measurements between sessions was determined using the CV, MDC and 95% LoA statistics. When using the mean of three measurements, CVs ranged from 5 to 16%, the MDC ranged from 24.52 to 91.01 N and the 95% LoAs ranged from -84.83 to 101.40 N. When using the median of three measurements, CVs ranged from 3 to 17%, the MDC ranged from 25.50 to 70.12 N and the 95% LoAs ranged from -68.16 to 73.55 N.

The only region to display a significant mean difference between sessions was the lesser toes (*p *= 0.01 when using the mean of three measurements and *p *= 0.03 when using the median of three measurements), where the percentage differences for the mean and median were both 10%.

### Inter-session reliability - peak pressure

The relative reliability between sessions when using the mean of three measurements was poor to moderate (ICCs between 0.51 and 0.72) for the total area, heel, midfoot, and MPJ345, and good (ICCs between 0.75 and 0.82) for MPJ1, MPJ2, hallux and the lesser toes. The relative reliability between sessions when using the median of three measurements was poor to good for the midfoot and hallux (ICCs of 0.54 and 0.72, respectively) and good (ICCs from 0.75 to 0.85) for the total area, heel, MPJ345, MPJ2, MPJ1, and the lesser toes (Table 5).

**Table 5:** Inter-session reliability of peak pressure (kPa).

	Mean of three trials					
**Region**	**Session 1** **mean (SD)**	**Session 2** **mean (SD)**	**ICC (95% CI)**	**CV (%)**	**MDC**	**95% LoA**

Total	2794.89 (382.45)	2726.24 (254.97)	0.58 (0.28 - 0.75)	7.6	549.17	-519.75 - 647.23
Heel	2500.69 (353.03)	2569.34 (509.94)	0.65 (0.39 - 0.82)	10.1	657.04	-774.72 - 647.23
Midfoot	912.01 (372.65)	843.37 (343.23)	0.72 (0.49 - 0.86)	21.5	519.75	-460.91 -588.39
MPJ345	2196.68 (362.84)	2245.72 (451.10)	0.51 (0.19 - 0.74)	12.9	774.72	-843.37 - 745.30
MPJ2	2637.98 (343.23)	2598.76 (362.84)	0.75 (0.54 - 0.87)	6.8	480.52	-451.10 - 529.55
MPJ1	1627.90 (441.29)	1735.77 (441.29)	0.87 (0.57 - 0.88)	12.5	578.59	-686.46 - 480.52
Hallux	1833.84 (421.68)	1775.00 (411.87)	0.78 (0.59 - 0.89)	10.8	539.36	-480.52 - 608.01
Lesser toes	990.47 (284.39)	1019.89 (372.65)	0.82 (0.65 - 0.91)	14.1	362.84	-421.68 - 362.84

	**Median of three trials**					

Total	1117.95 (186.32)	1186.60 (245.16)	0.81 (0.64 - 0.90)	3.1	225.55	-225.55 - 245.16
Heel	1314.09 (245.16)	1343.51 (225.55)	0.82 (0.66 - 0.91)	7.3	490.33	-509.94 - 578.59
Midfoot	500.13 (166.71)	500.13 (147.09)	0.54 (0.23 - 0.75)	23.3	558.97	-608.01 - 519.75
MPJ345	1274.86 (323.61)	1294.47 (254.97)	0.75 (0.54 - 0.87)	9.2	549.17	-529.55 - 588.39
MPJ2	1794.61 (382.45)	1824.03 (274.58)	0.78 (0.59 - 0.89)	7.3	529.55	-480.52 - 578.59
MPJ1	1127.76 (264.77)	1098.34 (225.55)	0.85 (0.72 - 0.93)	13.7	617.81	-608.01 - 647.23
Hallux	1049.31 (1255.25)	1059.11 (245.16)	0.72 (0.49 - 0.86)	10.7	519.75	-421.68 - 617.81
Lesser toes	558.97 (147.09)	578.59 (166.71)	0.79 (0.60 - 0.89)	16.8	441.29	-480.52 - 411.87

When using the mean of three measurements, CVs ranged from 6 to 22%, the MDC ranged from 362.84 to 774.72 kPa and the 95% LoAs ranged from -843.37 to 745.30 kPa. When using the median of three measurements, CVs ranged from 3 to 23%, the MDC ranged from 225.55 to 617.81 kPa and the 95% LoAs ranged from -608.01 to 647.23 kPa.

There were no systematic differences in mean values as evidenced by paired *t-*tests for any of the regions assessed.

### Inter-session reliability - average pressure

The relative reliability between sessions when using the mean of three measurements was good (ICCs between 0.75 and 0.88) for all regions except the midfoot, which displayed poor reliability (ICC 0.44). The relative reliability between sessions when using the median of three measurements was moderate for MPJ345, MPJ2, and the lesser toes (ICCs between 0.69 and 0.71) and good for the total area, heel, midfoot, MPJ1 and the hallux (ICCs between 0.77 and 0.88) (Table 6).

**Table 6:** Inter-session reliability of average pressure (kPa).

	Mean of three trials					
**Region**	**Session 1** **mean (SD)**	**Session 2** **mean (SD)**	**ICC (95% CI)**	**CV (%)**	**MDC**	**95% LoA**

Total	1137.57 (196.13)	1176.79 (215.74)	0.84 (0.68 - 0.92)	7.4	235.35	-264.77 - 205.93
Heel	1343.51 (215.74)	1333.70 (245.16)	0.82 (0.65 - 0.91)	7.3	264.77	-264.77 - 274.58
Midfoot	500.13 (127.48)	529.55 (147.09)	0.44 (0.10 - 0.69)	20.0	284.39	-313.81 - 254.97
MPJ345	1255.25 (196.13)	1265.05 (196.13)	0.75 (0.55 - 0.88)	7.8	274.58	-284.39 - 264.77
MPJ2	1755.39 (254.97)	1745.58 (264.77)	0.78 (0.58 - 0.59)	6.9	333.42	-333.42 - 343.23
MPJ1	1117.95 (245.16)	1117.95 (245.16)	0.88 (0.76 - 0.94)	7.7	235.35	-245.16 - 235.35
Hallux	1049.31 (245.16)	1059.11 (245.16)	0.75 (0.55 - 0.88)	11.5	333.42	-353.03 - 313.81
Lesser toes	578.59 (147.09)	578.59 (147.09)	0.81 (0.64 - 0.90)	11.2	176.51	-176.51 - 186.32

	**Median of three trials**					

Total	2716.44 (254.97)	2706.63 (225.55)	0.88 (0.76 - 0.94)	8.3	245.16	-333.42 - 196.13
Heel	2432.04 (372.65)	2441.85 (382.45)	0.78 (0.59 - 0.89)	7.5	274.58	-304.00 - 245.16
Midfoot	843.37 (392.26)	882.59 (402.07)	0.75 (0.53 - 0.87)	21.5	294.19	-304.00 - 294.19
MPJ345	2167.26 (343.23)	2196.68 (372.65)	0.69 (0.44 - 0.84)	11.4	382.45	-431.49 - 392.26
MPJ2	2647.79 (353.03)	2598.76 (333.42)	0.69 (0.44 - 0.84)	8.6	382.45	-460.91 - 402.07
MPJ1	1637.71 (451.10)	1657.32 (480.52)	0.77 (0.57 - 0.88)	8.5	254.97	-235.35 - 284.39
Hallux	1814.23 (382.45)	1716.16 (392.26)	0.77 (0.56 - 0.88)	12.9	372.65	-392.26 - 362.84
Lesser toes	970.85 (274.58)	931.63 (313.81)	0.71 (0.47v0.85)	12.6	196.13	-225.55 - 176.51

When using the mean of three measurements, CVs ranged from 6 to 20%, the MDC ranged from 176.51 to 333.42 kPa and the 95% LoAs ranged from -353.03 to 343.23 kPa. When using the median of three measurements, CVs ranged from 7 to 21%, the MDC ranged from 196.13 to 382.45 kPa and the 95% LoAs ranged from -460.91 to 402.07 kPa.

There were no systematic differences in mean values as evidenced by paired *t*-tests for any of the regions assessed.

## Discussion

Information elicited from the analysis of plantar pressures and forces during walking can be an integral component in the formulation of patient intervention plans [13]. Therefore, it is necessary to ensure that measurement systems, such as the TekScan MatScan^® ^which are commonly employed in the research and clinical setting, can accurately capture and reproduce plantar pressure measures of dynamic foot function on different occasions.

Intra-session repeatability was assessed for the three variables of interest by calculating CVs between three trials captured in a single session. The intra-session CVs for the seven analysed regions ranged from 3 to 22% for maximum force, 4 to 32% for peak pressure and 6 to 27% for average pressure. The midfoot and lesser toe regions displayed the greatest percentage differences for all three variables, which is consistent with previous reports using the Novel EMED^® ^plantar pressure platform [19] and indicates that these regions of the foot may be subject to inherent variability during gait. However, the CV for the total foot region for all three variables was relatively low (maximum force: 3%; peak pressure: 4%; average pressure: 6%). It can therefore be concluded that while the total force and pressure under the foot is relatively stable between repeated trials within the same session, there is greater variability within different regions of the foot.

Relative reliability was generally very high, with most ICC values greater than 0.70. Maximum force was shown to be the most reliable variable compared to peak pressure and average pressure. The two different calculation methods (the mean and median values of three trials) displayed moderate to good reliability for the variable maximum force throughout all seven regions (ICCs ranging from 0.71 to 0.97), whereas peak pressure and average pressure values were somewhat lower displaying poor to moderate reliability (ICCs ranging from 0.51 to 0.87, and 0.44 to 0.84, respectively). With some exceptions, taking the median of three trials, as opposed to taking the average of three trials generally resulted in slightly higher ICC values for all three variables. This may possibly be attributed to median values not being influenced by outliers, thus yielding a more reliable outcome. Therefore, the authors recommend the use of the median value in place of the mean value in future studies using the TekScan MatScan^® ^system.

Assessment for systematic differences between sessions indicated that maximum force in the lesser toes region exhibited a significant mean difference between sessions for both average and median calculations (*p *= 0.01 and *p *= 0.03, respectively). However, Cohen's *d *calculations indicated only a relatively small effect (*d *= 0.23 and a small percentage decrease of 10% for the mean, and *d *= 0.19 and a small percentage increase of 10% for the median). The remainder of the seven regions across all three variables did not display any systematic differences in mean or median values when captured one week apart.

Findings from this study assessing the TekScan MatScan^® ^system are in agreement with those reported by Gurney et al. [19] who assessed the reliability of the Novel EMED-at^® ^plantar pressure platform. Gurney et al. [19] conducted a between-day study protocol (5 separate days) assessing the reliability of nine asymptomatic participants for 10 regions of the foot for the variables of peak pressure, maximum force, impulse and contact time. The study concluded that areas of relatively high loading, such as the forefoot, showed higher reliability (ICC >0.90) than areas of lesser loading, such as the medial midfoot, which displayed lower reliability (ICC < 0.80). The Novel EMED-at^® ^platform is similar to the TekScan MatScan^® ^system, but has a slightly higher resolution of 2 sensors/cm^2 ^in comparison to 1.4 sensors/cm^2 ^and a slightly greater sampling frequency of 50 Hz in comparison to 40 Hz.

There are several limitations of this study that need to be considered when interpreting the findings. First, healthy young participants were recruited, so the reliability of these measurements cannot necessarily be generalised to other clinical populations. Confounding variables such as pain in symptomatic populations may have a significant impact upon the reproducibility of plantar measurements taken one week apart. Second, unlike the Novel EMED^® ^system, which uses automated software to apply the masks during data analysis, the TekScan MatScan^® ^requires a mask to be manually constructed and applied to the plantar pressure outputs for each individual participant. The standardised mask (Figure 1) could be altered in accordance to foot size and positioned with reference to the three foot regions (rearfoot, midfoot and forefoot) and anatomical landmarks (metatarsophalangeal joints, hallux and lesser digits). Although the mask template for each participant is saved and reapplied to subsequent trials, there is some potential for error resulting from different positioning of the foot between trials, thereby necessitating adjustment of the mask template upon application. This may affect the reliability of measurements [41]. Third, the relatively low sampling frequency (40 Hz) of the TekScan MatScan^® ^system makes this apparatus suitable for assessing walking trials only. Due to the low sampling rate it has the potential to inaccurately capture true peak data from more vigorous activities such as running. Fourth, although the relatively small size of the TekScan MatScan^® ^makes it portable and convenient it is unable to record consecutive steps and is limited to capturing only one plantar pressure recording, of either the left or right foot during each trial. Fifth, the discovery of the median value being reported to be more reliable than that of the mean could suggest that the sensor capabilities of this system are limited. Therefore, the performance characteristics of the plantar pressure mat sensors may be undesirable and should be interpreted with caution [42]. Sixth, previous work has shown that while the two-step gait initiation protocol we used provides similar forefoot peak pressure values to those obtained with the midgait protocol, rearfoot loading is reduced [43]. As such, rearfoot loading parameters need to be interpreted with some caution when using the two step protocol. Finally, the system under review is predominantly used to assess barefoot walking. Therefore it may be more suitable to implement an in-shoe pressure measurement system to assess plantar pressures associated with interventions such as insoles or therapeutic footwear.

Future investigations should now explore differences in plantar pressures and forces in a variety of other foot pathologies with consideration of the reliability values obtained in this study. The authors now intend to use the TekScan MatScan^® ^measurement apparatus to assess changes in plantar pressures and forces in people with hallux limitus/rigidus following treatment [44].

## Conclusion

The results of this study indicate that the TekScan MatScan^® ^system is a reliable instrument for assessing plantar forces and pressures during barefoot level walking in healthy participants taken one week apart. The system generally displayed moderate to good reliability for the three analysed variables of maximum force, peak pressure and average pressure throughout all seven assessed regions, with the exception of the mean average pressure value for the midfoot. Given the slightly higher reliability obtained from using the median compared to the mean of three repeated trials, the authors suggest that the median value is used for analysis. Overall, the TekScan MatScan^® ^system was found to exhibit similar reliability to other commercially available plantar pressure measurement systems and is suitable for use in the clinical and research setting.

## Competing interests

GVZ has no competing interests to declare. HBM is Editor-in-Chief and SEM is Assistant Editor of the Journal of Foot and Ankle Research. It is journal policy that editors are removed from the peer review and editorial decision-making processes for papers they have authored or co-authored.

## Authors' contributions

GVZ, HBM and SEM all conceived and designed the study. GVZ collected and analysed the data. GVZ drafted the manuscript with the assistance of both HBM and SEM. All three authors approved the final manuscript.
